# Molecularly Imprinted Electrochemical Sensor-Based Fe_2_O_3_@MWCNTs for Ivabradine Drug Determination in Pharmaceutical Formulation, Serum, and Urine Samples

**DOI:** 10.3389/fbioe.2021.648704

**Published:** 2021-04-08

**Authors:** Fatehy M. Abdel-Haleem, Eman Gamal, Mahmoud S. Rizk, Adel Madbouly, Rasha M. El Nashar, Badawi Anis, Hussam M. Elnabawy, Ahmed S. G. Khalil, Ahmed Barhoum

**Affiliations:** ^1^Chemistry Department, Faculty of Science, Cairo University, Giza, Egypt; ^2^Cairo University Centre for Environmental Hazards Mitigation, Environmental Studies and Research, CHMESR, Cairo University, Giza, Egypt; ^3^Spectroscopy Department, National Research Centre, Giza, Egypt; ^4^Environmental and Smart Technology Group (ESTG), Faculty of Science, Fayoum University, Faiyum, Egypt; ^5^Materials Science and Engineering Department, School of Innovative Design Engineering, Egypt-Japan University of Science and Technology (E-JUST), New Borg El-Arab City, Egypt; ^6^NanoStruc. Research Group, Chemistry Department, Faculty of Science, Helwan University, Cairo, Egypt; ^7^National Centre for Sensor Research, School of Chemical Sciences, Dublin City University, Dublin, Ireland

**Keywords:** carbon paste electrodes, molecularly imprinted polymers, detection limit, formation constant, selectivity against interfering species, H-bonding complexing strength, reversibility, lifetime

## Abstract

Ivabradine hydrochloride (IVR) is a medically important drug because of its ability to lower the heart rate. Techniques reported for IVR determination were expensive, laborious, besides being of poor selectivity. In this study, iron oxide @ carbon nanotube (Fe_2_O_3_@MWCNTs) nanocomposite and molecularly imprinted polymer (MIP) were synthesized and used in the fabrication of carbon paste electrodes (CPEs) for the potentiometric detection of IVR in biological and pharmaceutical samples. CPEs of the best sensor were formulated from graphite (41 wt%) as a carbon source, MIP (3 wt.%) as an ionophore, Fe_2_O_3_@MWCNTs (5 wt%) as a modifier, and nitrophenyl octyl ether (NPOE, 51 wt.%) as a conductive oil so-called plasticizer. The best sensor exhibits a Nernstian slope (response) of 56 mV decade^–1^ within the IVR concentration range from 1.0 × 10^–3^ M to 9.8 × 10^–8^ M with high selectivity against interfering species (ascorbic, maltose, glucose, lactose, dopamine, glycine) over those reported earlier. The use of Fe_2_O_3_@MWCNTs together with MIP in the electrode formulation was found to improve the limit of detection (LOD) from 630 to 98 nM along with high reversibility, a short response time of 30 s, and a good lifetime of more than 2 weeks. The sandwich membrane (SMM) method was used to quantify the H-bonding complexing strength of the MIP binding sites for IVR with Log β_*ILn*_ = 11.33. The constructed sensors were successfully applied for the IVR determination in blood serum, urine, and commercial formulations (Savapran^®^) with high sensitivity.

## Introduction

Electrochemical sensors are typically used to estimate the concentrations of many analytes (ions, neutral species, and biological molecules) in different samples. To date, electrochemical biosensors are widely used to detect and monitor various drugs, antibodies, enzymes, receptors, peptides, lectins, proteins, and biomarkers in the different biological fluids (blood, serum, urine, saliva, tissue) (Labib et al., 2016; Rasouli et al., 2019). These sensors are analyte-selective and can transform analyte concentration into different analytical detection signals (Abdel-Haleem et al., 2018, 2019) as voltage, current, or conductance (Labib et al., 2016). Modification of the electrode’s composition and surface characteristics can extensively improve its performance and lifetime (Yu et al., 2012; Bai and Zhou, 2014; Haichao et al., 2018). Nanostructured materials, e.g., graphene nanosheets, carbon nanotubes, metals, metal oxides, conductive polymers, and molecularly imprinted polymers, represent current strategies with significant improvements on the functionality of the sensors (Dhand et al., 2011; Anantha-Iyengar et al., 2019; Belbruno, 2019).

Carbon-paste electrodes (CPEs) are one of the most common types of ion-selective electrodes. The electrode is typically made of graphite powder, ionophore for binding the analyte, pasting liquid (mineral oils), and other modifiers that can facilitate the interaction of the analyte ion in the measured solution with the electrode active surface by decreasing the electrode resistance, which in turn reduces the response time (Bakker et al., 1997). In electrochemistry, synthetic species such as chelating ionophores and molecularly imprinted polymers (MIP) are incorporated typically as recognition elements; natural biomolecules such as DNAs, antibodies, receptors, enzymes, antibodies, and proteins are employed in biosensors. The CPEs can be used in voltammetry and amperometry; however, CPEs are also relevant in potentiometry. Also, potentiometry is advantageous over voltammetry, amperometry, and other electrochemical techniques in terms of its simplicity, no need for sophisticated instrumentation which makes it a cost-effective technique, application in colored and turbid solution, applicability in wide linear dynamic range from a tenth of molar concentration to nanomolar concentrations, and the fast response time so that it can be applied in the routine analysis (Bakker et al., 1997; Abdel-Haleem et al., 2020; El-Beshlawy et al., 2021). The composition of CPEs can be modified with different modifiers (e.g., graphene, MWCNTs, Au, TiO_2_, Ag, Pd, Fe_2_O_3_) for electrochemical detection of pharmaceutical compounds, pollutants, and biological molecules in the presence of the different interfering species (Prasad et al., 2018; Samyn et al., 2018; Gopalakrishnan et al., 2019; Abdel-Haleem et al., 2020; El-Beshlawy et al., 2021). Nanoparticles’ (NPs) involvement in the CPEs was found to have a set of remarkable improvement features on the response. NPs offer a higher surface area and excellent electrical conductivity (Baptista et al., 2015; Jeevanandam et al., 2018; Haichao et al., 2019). For example, modified CPEs based on graphene and MWCNTs were reported to have fast charge transfer and high electroactive surface area (Barhoum et al., 2018b; Karatutlu et al., 2018). Although preparation of some NPs is expensive as metal oxide NPs, other types of NPs are so facile that they can be prepared in cost-effective methods (Baptista et al., 2015; Barhoum et al., 2018b; Jeevanandam et al., 2018; Karatutlu et al., 2018; Prasad et al., 2018; Samyn et al., 2018; Gopalakrishnan et al., 2019; Haichao et al., 2019). One of these cost-effective methods is the preparation of the graphene oxide (GO) using the Hummer method and its modifications (Hummers and Offeman, 1958; Yu et al., 2016; Zaaba et al., 2017) and preparation of other carbonaceous NP materials such as graphene (Gr), reduced graphene oxide (rGO), and MWCNTs (Baptista et al., 2015; Barhoum et al., 2018b; Karatutlu et al., 2018; Prasad et al., 2018; Haichao et al., 2019). Concerning metal oxide NPs, iron oxide NPs can be prepared very easily with a very cheap method resulting in the oxide of different properties (Woo et al., 2004; Kostyukova and Chung, 2016). Surface modification of MWCNTs and graphene with metals and metal oxide NPs increases the number of receptor sites needed for biorecognition and accordingly increases the sensor’s affinity to interact specifically with the target analytes, with higher sensitivity (Hummers and Offeman, 1958).

Molecularly imprinted polymers (MIP) are macromolecular polymeric species that contain very specific recognition sites (molecular cavities) in the polymer matrix that it is specific and selective for the template analyte molecule (Yoshikawa et al., 2016). MIP can be synthesized by the physical interaction (non-covalent bonding) of a target molecule with functional monomers followed by selective extraction of the target molecule from the polymeric matrix rendering specific binding cavities (recognition sites) complementary in the shape and size to the targeted analyte (template) (Yoshikawa et al., 2016). MIPs were reported as highly selective and specific ionophores in electrochemical sensors, of low detection limits, extended usability time, and fast response time (Abdel Ghani et al., 2016; Abdel-Haleem et al., 2016; Yoshikawa et al., 2016; Anantha-Iyengar et al., 2019). The use of MIPs as ionophores in CPEs based on ion selectivity was also advantageous in terms of resistance to heat and pressure, high mechanical properties, inertness, low cost, ease of preparation, and insolubility in water and most organic solvents (Švancara et al., 2009; Yoshikawa et al., 2016).

Ivabradine HCl (IVR), FDA approved in 2015, is a drug recommended for a heart rate decrease, angina pectoris, and symptomatic chronic heart failure in beta-blocker-resistant patients or under max dose (Riccioni, 2012). The initiative dose of IVR is 2.5 mg twice daily; however, for patients with a heart rate of >60 beats/min, the dose might be increased to 7.5 mg (administered twice daily) (Seerapu and Srinivasan, 2010). Headache, uncontrolled blood pressure, severe and prolonged bradycardia, and blurred vision are common side effects encountered with high doses of IVR (Riccioni, 2012), and it is also not recommended in case of pregnancy due to possible fatal toxicity and teratogenicity (Seerapu and Srinivasan, 2010). Because of IVR medical importance and the requirement of overdose problem monitoring, several methods were proposed for its determination in the different samples such as chromatographic methods, spectrophotometric methods, and spectrofluorimetric methods (Patel et al., 2014). However, these techniques may offer poor selectivity besides being expensive and laborious.

Based on the literature, IVR was determined potentiometrically using PVC membrane electrodes incorporating sulfonated calixarene and hydroxypropyl-ß-cyclodextrin (Abo-Talib et al., 2015) which exhibited low to moderate selectivity. Yet, no MIP (ionophore) as ionophore has been reported for IVR determination. In this study, we report the developing carbon paste potentiometric sensor based on Fe_2_O_3_@MWCNTs as a carbon paste modifier, which was prepared with a cost-effective method. MIPs were used as selective recognition materials for IVR in physiological fluids (urine and serum) and pharmaceutical dosage formulation (Savapran^®^). The type and content of ionophore (MIP), plasticizer type (o-NPOE, TCP), carbon materials (graphite, MWCNTs, Fe_2_O_3_@MWCNTs), suitable pH range, and effect of interfering species (anions, cations, nanoionics) on the performance (sensitivity, selectivity, response time, reversibility) of the sensors were also studied. Four ratios of bulk MIPs were polymerized, and the one showing the highest binding strength (capacity) with IVR was chosen as a recognition ionophore in the construction of the CPEs. The binding strength between the ionophore as a host and the IVR analyte as a guest was estimated potentiometrically using the sandwich membrane method (SMM), for the first time in the case of MIP ionophore-based ion-selective electrode (ISEs). Besides, modification with MIP as ionophore improved the sensitivity and selectivity toward IVR in comparison to other previously reported IVR sensors due to the selective recognition properties of MIPs compared to other traditional modifiers (Abo-Talib et al., 2015; Abdel-Haleem et al., 2020). The matched potential method (MPM) was performed to ensure the results of the SSM and to measure the selectivity of neutral interfering ions that cannot be measured using the SSM.

## Experimental

### Chemical Reagents

**FIGURE 1:**
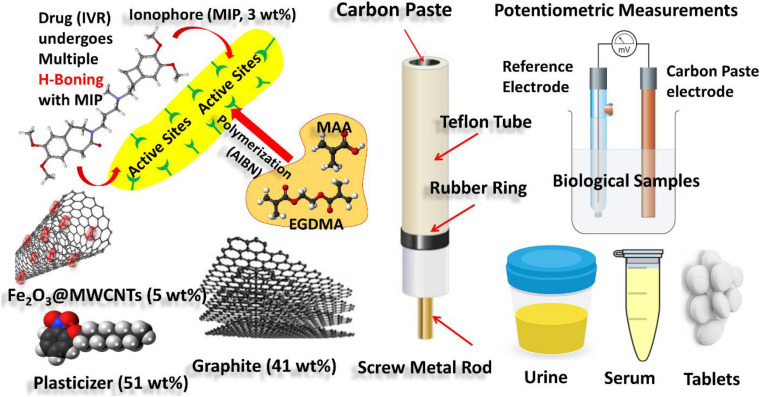
Schematic representation showing experimental work: (i) synthesis of MIPs of IVR drug-using MAA and EGDMA as building monomers and AIBN as initiator; (ii) CPE formulation using graphite as carbon source, MIP as an ionophore for the IVR drug, Fe_2_O_3_@MWCNTs (5 wt%) as a modifier, and NPOE as a plasticizer; (iii) electrochemical cell based on the modified CPE as working electrode and reference Calomel reference electrode connected to a Jenway 3310 pH meter of (England) for potentiometric determination of the IVR drug in biological fluid samples (urine and serum blood).

Ivabradine HCl drug was obtained from the National Organization for Drug & Control Research (Giza Governorate, Egypt). An IVR dosage form 5 mg/tablet (Savapran^®^, batch no. 964294) was purchased from the local market. Ferric nitrate non-ahydrate (Fe(NO_3_)_3_⋅9H_2_O, 99%), urea (CH_4_N_2_O, 99%), magnesium nitrate hexahydrate (Mg(NO_3_)_2_⋅6H_2_O, 99%), ammonium molybdate tetrahydrate [(NH_4_)_6_Mo_7_O_24_⋅4H_2_O, 99%], nitric acid (HNO_3_, 70%), sulfuric acid (H_2_SO_4_, 97%), and acetic acid (CH_3_COOH, 99%) were supplied from Sigma-Aldrich and used for the synthesis of the Fe_2_O_3_@MWCNT particles. Methacrylic acid (MAA, 99%), ethylene glycol dimethacrylate (EGDMA, 97%), 2,2′-azobisisobutyronitrile (AIBN, 98%), and dimethyl sulfoxide (DMSO, 98%). Graphite (Gr, 99%, <45 μM), potassium tetrakis[3,5-bis(trifluoromethyl)phenyl]borate (TPPB, 99%), tricresyl phosphate (TCP, 98%), 1-(2-nitrophenoxy)octane (NPOE, 99%), and dioctyl phthalate (DOP, 97.0%) were supplied from Sigma-Aldrich and used for the sensor’s construction, Figure 1. Acetone (97%, ADWIC), tetrahydrofuran (THF, 97%), glucose (99%), maltose (98%), sodium hydroxide (96%), absolute ethanol (99%), methanol (98%), hydrochloric acid (37%), sodium chloride (99%), aluminum chloride (97%), ammonium chloride (99%), magnesium chloride (98%), ferric chloride (97.5%), and potassium chloride (99%) were obtained from ADWIC (Cairo, Egypt) and used for the selectivity measurements.

### Synthesis of Molecularly Imprinted and Non-imprinted Polymers

Molecularly imprinted polymers (MIPs) of IVR were prepared as reported earlier (Abdel Ghani et al., 2016). Approximately 0.5 mmol of IVR and different ratios of MAA and EGDMA were mixed and in a screw-capped Pyrex tube as given in Table 1. Approximately 0.1 mmol in 2 mL DMSO of the initiator (AIBN) was gradually added, and the mixture was degassed for 5 min with pure Ar stream and left in an oil bath for 24 h at 60°C to assure complete polymerization (Figure 1). The produced MIPs were finely powdered using a pestle and mortar followed by sieving to <45-μm size (Abdel Ghani et al., 2016; Abdel-Haleem et al., 2016). For extraction of IVR, the MIP particles were dispersed (incubated) in (9:1, v/v) a methanol–acetic acid mixture for 20 h and then filtered and dispersed in methanol for 10 h and finally filtered and dispersed in DI water for 24 h. The removal of IVR (template) was studied by testing increments after each incubation step spectrophotometrically at 286 nm. The IVR-free MIP was then dried and kept at room temperature for further experiments. The non-imprinted polymer (NIP) was synthesized in the same method as the MIP powder particles without IVR addition (Abdel Ghani et al., 2016).

**TABLE 1:** The composition of the different non-printed (NIP) and molecularly imprinted (MIP) polymers, binding capacity (BC), imprinting factor (IF), and the mole ratio of IVR, MAA, and EGDMA in about 4 mol of DMSO that was used as a solvent in all ratios.

Polymer	Constituents				Response characteristics	
	IVR	MAA	EGDMA	Molar ratio	BC	IF
MIP1	0.5	2	10	1:4:20	0.0022	1.30
MIP2	0.5	2	20	1:4:40	0.0100	1.36
MIP3	0.5	3	20	1:6:40	0.0120	1.60
MIP4	0.5	4	20	1:8:40	0.0010	1.20
NIP1	–	2	10	0:4:20	0.0017	–
NIP2	–	2	20	0:4:40	0.0073	–
NIP3	–	3	20	0:6:40	0.0085	–
NIP4	–	4	20	0:8:40	0.0008	–

### Preparations of MWCNT and Fe_2_O_3_@MWCNT Nanocomposite

The chemical vapor deposition (CVD) route and Fe_1_Mo_1_MgO_13_ powder as catalysts were used for the synthesis of carbon nanotubes (MWCNTs), as reported earlier (Iconaru et al., 2016; Rahmawati et al., 2017). The catalyst (300 mg) was spread onto a ceramic boat and placed inside an electric tube furnace at 700°C for 30 min under 100 sccm H_2_ atmospheres. Acetylene (50 sccm) was then purged for 30 min, then the furnace was cooled to atmospheric conditions. The as-prepared MWCNTs were decorated with Fe_2_O_3_ NPs as reported elsewhere (Liao and Pan, 2011; Tucureanu et al., 2016). For the decoration of MWCNTs with Fe_2_O_3_ NPs, the MWCNT powder was then treated using HCl (37 wt%) at 60°C for 24 h under reflux (Rahmawati et al., 2017). Two hundred milligrams of MWCNTs was sonicated for 30 h in 12 mL of a mixture of concentrated HNO_3_ and concentrated H_2_SO_4_ (1:3). The obtained slurry was filtered, washed with deionized water until neutral. Around 50 mg MWCNT powder was added to 50 ml of a methanolic solution of Fe(NO_3_)_3_⋅9H_2_O with a weight ratio of 1:1. The slurry was first stirred for 60 min at room temperature, then the temperature was increased to 80°C until complete evaporation of methanol. Finally, the obtained Fe_2_O_3_@ MWCNT powder was treated with acetic acid vapors for 15 min followed by annealing at 450°C (under Ar atmosphere) for 30 min (Liao and Pan, 2011; Tucureanu et al., 2016).

### Characterization Techniques

A two-electrode setup cell, based on the modified CPE and reference Calomel reference electrode (Hanna-Italy), connected to a Jenway 3310 pH meter of (England), was applied for measurement of different IVR concentrations (10^–8^ to 10^–3^ M); the cell can be described as follows:

(1) Hg/HgCl2/2KCl(saturated)//samplesolution//CPE

UV-Vis spectroscopic measurements were performed using an OPTIZEN POP-automated UV-Vis spectrophotometer (Korea). MIP incubation with IVR solutions was performed in an Eppendorf Thermomixer comfort (Germany). Shaking was performed by a programmable incubator/mixer (Awareness Technology Inc., United States). Centrifugation was performed using the spectra scientific Merlin-503 centrifuge (England). A JEOL 1011 transmission electron microscope was used for sample imaging (Barhoum and Luisa García-Betancourt, 2018; Barhoum et al., 2018a). A SHIMADZU IR spectrometer was employed for Fourier-transform infrared spectroscopy (FTIR) analysis non-imprinted, MIP, and NIP (Teng et al., 2003; Yan et al., 2013).

### Equilibrium Binding Assay

Binding efficiency of the prepared IVR-MIP samples was tested through incubation with shaking approximately 20 mg of the prepared MIPs or NIPs with 5 mL of different aqueous IVR solutions (10^–4^, 10^–5^, and 10^–6^ M) in a falcon tube, for 2 h at 25°C. The mixture was centrifuged at 14,000 rpm for 15 min and then filtered using a 0.22-mm syringe filter. The unbound IVR concentration in the supernatant was measured spectrophotometrically at 286 nm. The binding capacity of IVR to the polymer can be estimated through the subtraction of the unbound concentration from the initial incubated, per each of the tested concentrations (Abdel Ghani et al., 2016). The binding capacity (B, μmol g^–1^) for MIP and NIP can be calculated as:

(2) $$\text{B}=\frac{\left(C\,i-C\,f\right)\,V}{M}$$

where

C_*i*_ is the initial IVR concentration (mM)

C_*f*_ is the remaining unbound IVR concentration after adsorption (mM)

V(mL) is the portion volume, and M (g) is the mass of the NIP or MIP used.

The imprinting factor (IF) was then calculated by comparing the values of the binding capacity of the MIPs to that of its equivalent NIPs (Abdel Ghani et al., 2016), as follows:

(3) $$\text{IF}=\frac{B\,\left(M\,I\,P\right)}{B\,\left(N\,I\,P\right)}$$

### Electrode and Standard Solution Preparation

Carbon-paste electrodes were prepared with different compositions of graphite and a plasticizer (TCP or NPOE) together with the different tested modifiers, namely, the ionophore (MIP3 or NIP3) and modifier (MWCNTs and Fe_2_O_3_@MWCNTs), for the carbon paste (Abdel-Haleem et al., 2018). Both TCP and NPOE are highly lipophilic plasticizers, and thus, they were used in the fabrication of polymer membranes for ion-selective electrodes. The modified carbon pastes were packed in the Teflon electrodes (hole of 0.7 cm diameter, 12 cm length, 0.35 cm deep), as shown in Figure 1. The modified CPE surface is usually polished before use onto a smooth paper till shiny before dipping in a series (10^–8^ to 10^–3^ M) of dipped-in IVR concentration series (10^–8^ to 10^–3^ M) to obtain the calibration curve (Abdel-Haleem et al., 2018). The linear dynamic range is the concentration ratio of the upper and lower detection limits, where the electrode responds in the Nernstian way (Lrving et al., 1976; Bakker et al., 1997). The detection limit was calculated from the intersection of the extrapolation of the linear segments at the lower concentration limit (Lrving et al., 1976); this method was recommended by IUPAC (Lrving et al., 1976; Bakker et al., 1997).

### Selectivity Against Interfering Species

The electrode selectivity was tested against different interfering species (ascorbic, maltose, glucose, lactose, dopamine, glycine) for 10^–3^ M solutions of IVR and other tested species by the separate solution method (SSM) (Bakker et al., 1997), and the selectivity coefficient against interfering species, ${\text{K}}_{\text{IVR},J^{\text{Z}+}}^{\text{pot}.}$, was calculated by Eq.3:

(4) KIVR,JZ+pot.=(E-2E/1S)+log[IVR]-log[J]Z+/Z1

where

E_1_ and E_2_ are the measure potentials for the cell in 10^–3^ M IVR solution

J^*Z+*^ interfering cation with a charge of Z

S is the slope of the IVR calibration curve.

The matched potential method (MPM) (Abdel Ghani et al., 2016; Abdel-Haleem et al., 2019) was also used for the determination of potentiometric selectivity coefficients of carbon paste electrodes using Eq. 4:

(5) $${\text{K}}_{{\text{J}}^{+\text{z}}}^{\text{pot}}=\frac{{\text{a}}_{\text{ii}}-{\text{a}}_{\text{i}}}{{\text{a}}_{\text{J}}}$$

### Formation Constant Estimation

A PVC membrane was prepared by using TPPB as an ion exchanger and TCP as a plasticizer (membrane I), Table 1. Another membrane was prepared using the same previous components and either MIP3 or NIP3 (membrane II) of total graphite of weight 0.3 g dispersed in 2 mL of THF and air-dried for 24 h. Sandwich membranes were fabricated by pressing the two individual membranes (membrane I) and (membrane II) over each other (Abdel-Haleem et al., 2019) before being glued to the end of the PVC tubes using a PVC/THF mixture; the resulting combined membrane was then allowed to air-dry for 10 min. An IVR solution (10^–4^ M) was used as filling and 24-h soaking solutions before the first solution.

The hydrogen bonding strength of the MIP interaction with IVR was estimated, and the formation constant β_*IL_n*_ can be calculated from Eq. 5:

(6) βI⁢Ln=(LT-n⁢RTZI)⁢e-n⁢x⁢p⁢(EM⁢ZI⁢FR⁢T)

where *L*_*T*_ is the MIP concentration in the membrane, *R*_*T*_is the concentration of TPPB, *n* is the IVR–ionophore stoichiometric ratio, *R* is the gas constant, and *T* and *F* are the absolute temperature and Faraday constant, respectively (Abdel-Haleem et al., 2019). *Z*_*I*_is the charge of ion I.

### Determination of IVR in Savapran^®^ and Spiked Biological Samples

A series that consists of 10^–4^, 10^–5^, and 10^–6^ M in IVR was prepared from an equivalent amount of Savapran^®^ tablets (ground powder) dissolved in deionized water and filtered using a syringe filter (Abdel-Haleem et al., 2018). The obtained solutions were tested using the best sensor, and the obtained results were compared to those given by the high-performance liquid chromatography (HPLC), the method reported in Nowakowska et al. (2017). For spiking of biological samples, 4 mL urine or 1 mL serum was mixed with different amounts of IVR and completed with distilled water to 25 mL for preparing 50.5, 5.05, and 0.50 μg/mL IVR solutions (Abdel Ghani et al., 2016; Abdel-Haleem et al., 2018).

## Results and Discussion

### Morphological Characteristics of Fe_2_O_3_@MWCNTs

Pristine MWCNTs were synthesized by the CVD followed by decoration with Fe_2_O_3_ NPs using wet combustion synthesis. The MWCNTs and Fe_2_O_3_@MWCNTs (Barhoum et al., 2019) were then used as modifiers for the carbon paste electrodes. The use of magnetic Fe_2_O_3_ NPs in electrochemical sensors was found to offer unique properties over other strategies employing non-magnetic NPs in terms of enhancing the sensor sensitivity, improving the limit of detection (LOD), and shortening the time of analysis (Rocha-Santos, 2014).

**FIGURE 2:**
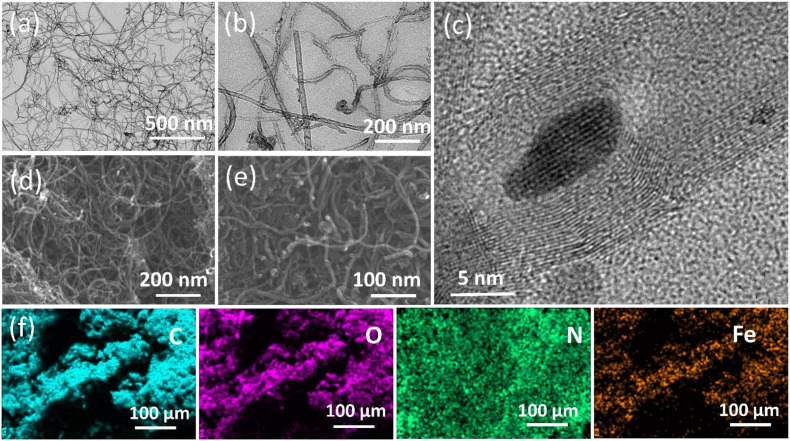
TEM images of the pristine MWCNTs **(a)** and Fe_2_O_3_@MWCNTs **(b,c)**. SEM images of the pristine MWCNTs **(d)** and Fe_2_O_3_@MWCNTs **(e)**. SEM-EDX elemental mapping **(f)** for carbon, oxygen, nitrogen, and iron of the Fe_2_O_3_@MWCNT nanocomposite particles.

SEM and TEM were employed (Figure 2) to study the morphology and elemental composition of the as-prepared unmodified MWCNT and modified Fe_2_O_3_@MWCNT samples. SEM and TEM images (Figures 2a,b) show that the prepared MWCNTs have a length of several micrometers. The HRTEM images (Figure 2c) show that the MWCNTs have about 20 well-graphitized walls and are decorated with ∼10 nm Fe_2_O_3_ NPs. The fact that these NPs consist of Fe_2_O_3_, purified MWCNTs, and Fe_2_O_3_@MWCNTs nanocomposite was further examined by XRF, SEM-with elemental mapping, and XRD.

The XRF analysis indicates that the purified MWCNTs are made of 99.8% carbon with traces of Fe, Mn, Mo, Si, and Cl. The carbon content of Fe_2_O_3_@MWCNTs is about 99.1% while the percentage of Fe_2_O_3_ is ∼0.7 wt% with minor contents of Mn, Ca, Mo, Si, Cl, and S. The SEM-EDS with mapping showed that the pristine MWCNTs are made of 96.15% carbon, 1.33% oxygen, and 2.52% nitrogen as an atomic percent. The Fe_2_O_3_@MWCNTs are made of 89.5% carbon, 4.5% oxygen, 2.7% nitrogen, and 3.3% iron as an atomic percent.

**FIGURE 3:**
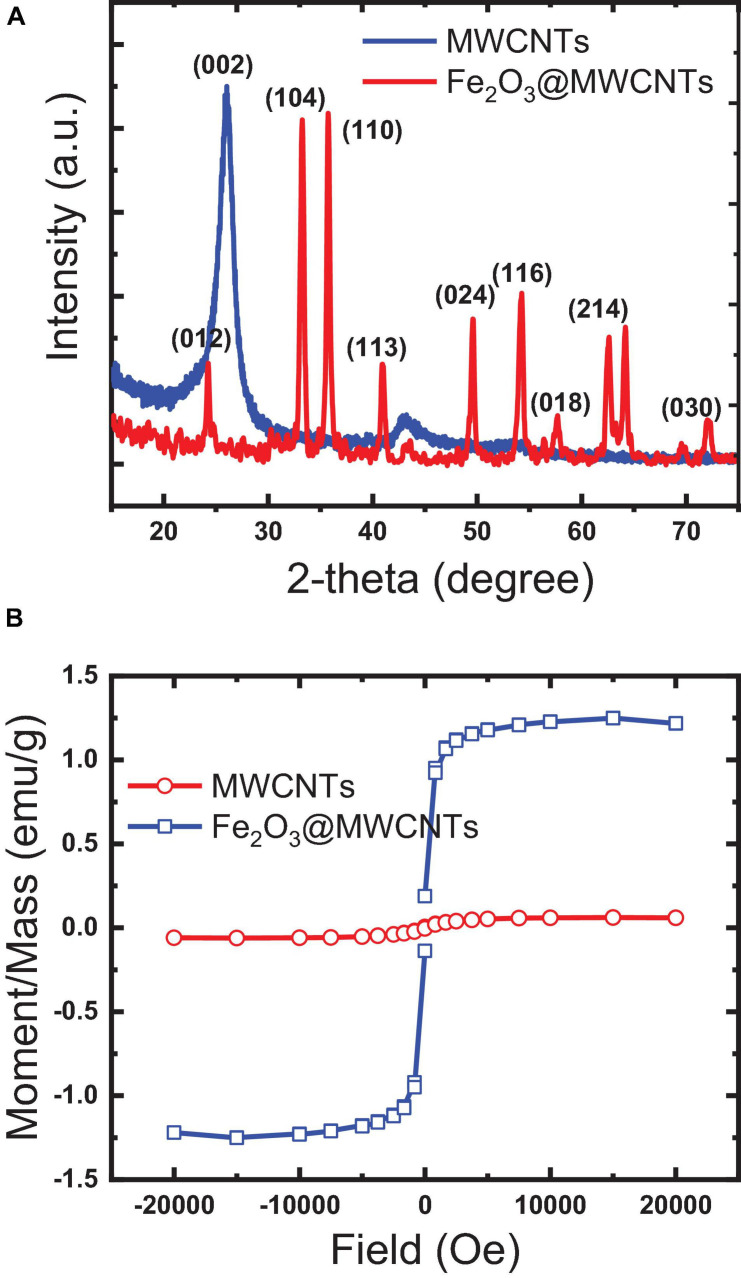
Characteristics of the papered pristine MWCNTs and Fe_2_O_3_@MWCNTs: **(A)** X-ray diffraction; **(B)** magnetism.

XRD analysis in Figure 3A shows a typical spectrum of MWCNTs and Fe_2_O_3_@MWCNTs, which confirms the SEM and TEM results. The first intense peak at around 26° is that the MWCNT peak belongs to the crystalline peak (002) of the hexagonal graphite structure (Fahmi and Chang, 2014). The second peak at approximately 42° is the (100) peak of the MWCNT structure. The resulting pattern was compared to the standard MWCNT and Fe_2_O_3_@MWCNT-XRD patterns, and it was found that the cubic crystalline structure of maghemite Fe_2_O_3_ (JCPDS 04-015-9580) is a major difference. The characteristic peak at 26° attributed to the plane (002) of MWCNTs while other diffraction peaks at 35.6°, 43.15°, 53.28°, 57.3°, and 63.12° are attributed to planes (311), (400), (422), (511), and (440) of the Fe_2_O_3_ phase.

The magnetization of MWCNTs through the decoration with Fe_2_O_3_ was studied by VSM (Iconaru et al., 2016; Rahmawati et al., 2017). Figure 3B illustrates the magnetic hysteresis loops of as-synthesized pristine MWCNTs and Fe_2_O_3_@MWCNTs. The saturation magnetization of the MWCNTs and Fe_2_O_3_@MWCNTs reaches 0.05emu/g and 1.4 emu/g, respectively. This low value of magnetization for Fe_2_O_3_@MWCNTs in comparison with that of MWCNTs can be related to the low Fe_2_O_3_ contents which were found to be around 0.55 wt%, as determined using XRF.

**FIGURE 4:**
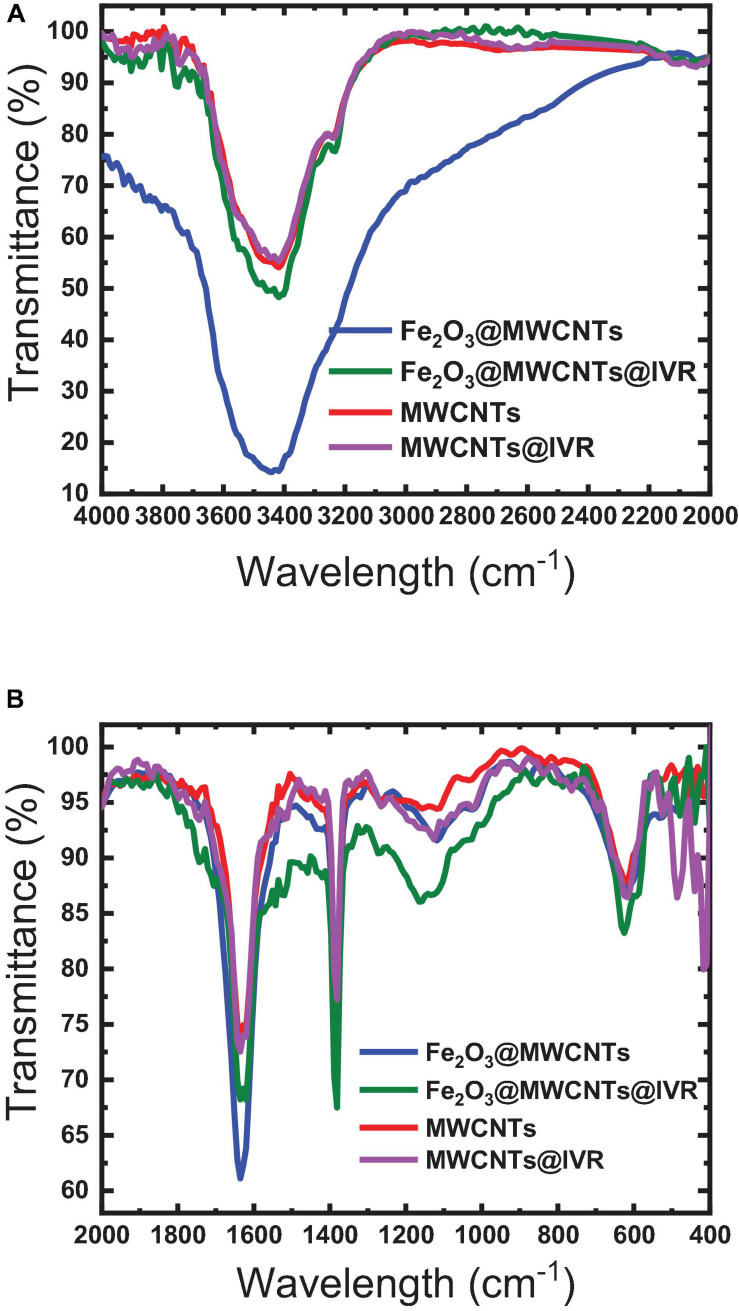
FTIR spectra of the MWCNTs and Fe_2_O_3_@MWCNTs before and after interaction with IVR. **(A)** 4000–2000 cm^−1^; **(B)** 2000–400 cm^−1^.

The FTIR spectrum of Fe_2_O_3_ is found to have a broad IR peak around 542 cm^–1^ and the narrow peak at 433 cm^–1^ corresponding to the bonds in Fe_2_O_3_ formation, while the FTIR spectrum of MWCNTs exhibited peaks at 817, 949, and 1,057 cm^–1^ which can be attributed to the nitrogen defects in the MWCNT structure, υ (C = C) of MWCNT backbone, and υ (C-O) of MWCNT carboxyl groups (Liao and Pan, 2011; Tucureanu et al., 2016). These peaks were shifted to 810, 933, and 1,072 cm^–1^ in the Fe_2_O_3_@MWCNT spectrum (Figure 4B), which confirms the interaction between the MWCNTs and Fe_2_O_3_ NPs. This interaction can be ensured also by the broadening of the peak at 3,400 cm^–1^ in the MWCNT spectrum after interaction with iron NP as shown in the Fe_2_O_3_@MWCNT spectrum (Figure 4A). This agrees with the previously reported results of Yan et al. (2013) and Teng et al. (2003) for composite Fe_2_O_3_@MWCNT nanoparticles prepared with sequential analysis. The FTIR of Fe_2_O_3_@MWCNTs exhibited peaks at 3441 cm^–1^ which were shifted to 3417 cm^–1^ upon the formation of hydrogen bonding with IVR analyte (Figure 4). Thus, it is also worth emphasizing herein that MWCNTs can interact with IVR through stacking π–π interactions to form supramolecular complexes (Mehra and Palakurthi, 2016; Chen et al., 2018). Masotti and Caporali (2013) reviewed several studies that confirm the existence of different types of interaction between MWCNTs and magnetic NPs such as electrostatic, π–π stacking, and hydrophobic interactions (Masotti and Caporali, 2013). They reported that MWCNTs decorated with magnetic NPs could be applied for magnetic data storage, for heterogeneous catalysis, and in electronic devices.

### Binding Capacity, Imprinting Factor of MIPs, and Interaction Mechanism

Molecular imprinting technology enhances the ability of the as-prepared MIPs to bind to the IVR molecules at different binding sites such as -N, -C-H, -O-CH_3_, or -C = O groups (Abdel Ghani et al., 2016; Abdel-Haleem et al., 2016; El Nashar et al., 2017; Anantha-Iyengar et al., 2019). The total amount of binding sites and their distribution is not well known. However, the higher the similarity in chemical structure between IVR and the cavities in MIP, the stronger the selective complexation. Typically, the molar ratio of (monomer MAA: cross-linker EGDMA: template IVR) affects significantly the number and configuration of the binding sites and their binding probability with IVR molecules (Abdel Ghani et al., 2016). Increasing the molar ratio of MAA: EGDMA from MIP1 to MIP3 enhanced the monomer–template interactions increasing the imprinting factor (IF) of MIP3 (1.60) compared to MIP1 (1.30) (Table 1). Yet, it was noticed that a further increase of MAA:EGDMA ratio, as in MIP4, caused an increase in the polymer rigidity leading to retardation of the polymer/template interactions, resulting in a decrease in IF (Abdel Ghani et al., 2016; Abdel-Haleem et al., 2016; Belbruno, 2019). Accordingly, MIP3 and NIP3 were chosen for all the subsequent sensor studies.

**FIGURE 5:**
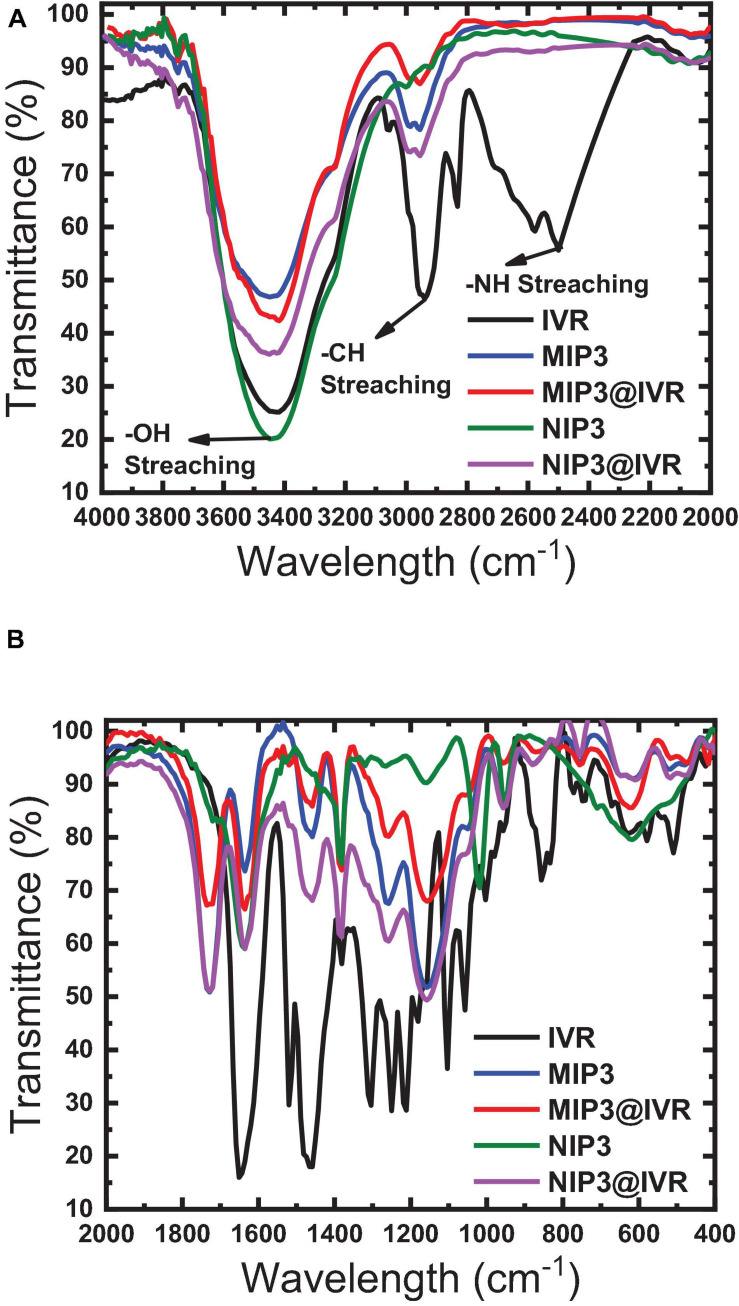
FTIR spectra of MIP3, NIP3, and IVR before (leached) and after interaction (unleached) in ranges **(A)** 2,000–4,000 cm^–1^ and **(B)** 400–2,000 cm^–1^.

A higher binding capacity observed for MIP compared to NIP indicates that the specific binding sites that are available on the as-prepared MIP particles will lead to higher binding efficiency, lower the detection limit, and improve the selectivity toward different molecules or ions that can be present in the tested matrix. The binding sites inside MIP cavities are thought to interact with the drug (IVR) molecules through non-covalent interactions based on dispersive forces, ion pairing, and hydrogen bonds (Abdel Ghani et al., 2016; Abdel-Haleem et al., 2016; Yoshikawa et al., 2016). Interfering substances can be sterically excluded from binding to the imprinted cavities due to size limitations. Non-specific interactions may also occur, where IVR molecules may also be adsorbed on the surface of the MIP or NIP particles or on places where there are no functional monomer units at all via hydrophobic interactions especially if the adsorption medium is partly aqueous (Abdel Ghani et al., 2016; Abdel-Haleem et al., 2016; Yoshikawa et al., 2016). The IR spectra of polymer ionophores before and after interaction with IVR (leached and un-leached polymer particles) were recorded as given in Figure 5 to prove that the major interaction between IVR and the different ionophores (MIP3 and NIP3) is resulting from the non-covalent hydrogen bonding (Lrving et al., 1976; Barhoum et al., 2018a). This suggestion was assisted using the peak at 1,649 cm^–1^ in IVR spectra corresponding to C = O which was shifted to 1,608 cm^–1^ after interaction with MIP3 or NIP3, which represents strong evidence of hydrogen bonding between both MIP3 and NIP3 with IVR (Figure 5B). Also, the H-bonding between IVR and MIP3 is evidenced by the shift in the O–H band from 3,448 cm^–1^ in MIP3 to 3,417 cm^–1^ after IVR interaction, and from 3,429 cm^–1^ in the case of NIP3 to 3,426 cm^–1^ after IVR interaction (Figure 5A). All these H-bonds between carbonyls of IVR and MIP3 carbonyls and nitrogen led to the high selectivity of MIP3.

**FIGURE 6:**
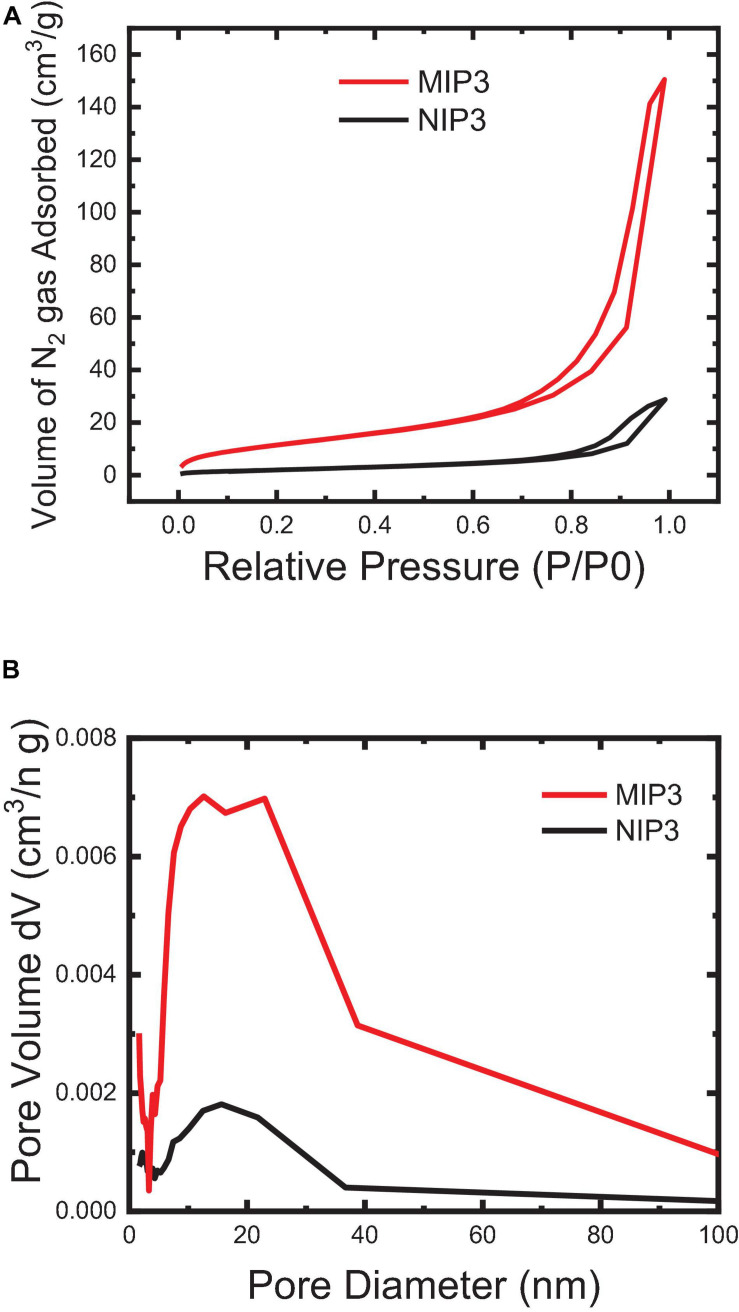
BET surface area of the MIP and NIP powder particles: **(A)** N_2_ Adsorption-desorption (BET isotherm) and **(B)** BJH pore size distribution.

The imprinting efficiency of the MIP particles compared to NIP can be characterized by using the BET nitrogen adsorption method. Generally speaking, the increase in the BET surface area and average pore volume and pore diameter of MIP particles than those of NIP indicates that the polymer possesses a higher accessibility and a better capacity for rebinding in its pores (Figure 6). The results showed that the specific BET surface area of MIP (57.51 m^2^/g) is relatively large and is more than four times that of NIP (13. 77 m^2^/g), and similarly, the total pore volume of MIP (0.239 cc/g) is significantly high as and about five times that of NIP (0.047 cc/g). The average pore diameter of NIP is relatively higher (12.6 nm), though that of MIP (15.6 nm) is enough for the free flow of the IVR molecules in the MIP matrix. Overall, the observed results from BET analysis indicated that the MIP particles could bind more efficiently than the NIP.

### Effect of the Electrode Composition

The effect of composition was studied primarily by changing the amount of MIP3 in sensors 1 and 2. It can be seen in Table 2 that increasing the ratio of MIP3 in sensor 2 caused a higher slope (44.0 mV/conc. decade) than that of sensor 1 (39.0 mV/conc. decade), which is expected to be due to the higher extent of binding. A further increase in the amount of MIP3 ionophore was found to affect the mechanical properties of the paste and make it hard to homogeneously mix its components.

**TABLE 2:** Effect of the carbon paste electrode composition on the response characteristics: slope concentration range (CR) and detection limit (DL).

Sensor	Electrode composition				Response characteristics			
	Graphite	Plasticizer	Ionophore	Modifier	Slope^*a*^		CR	DL
			
	Wt%	Wt%	Wt%	Wt%	mV/conc. decade	R^2^	M	M
1	49	50 TCP	1 MIP3	–	39.0 ± 1.3	0.975	10^–4^–10^–6^	1.0 × 10^–6^
2	47	50 TCP	3 MIP3	–	44.0 ± 1.1	0.983	10^–4^–10^–6^	1.0 × 10^–6^
3	43	54 NPOE	3 MIP3	–	52.0 ± 0.6	0.992	10^–4^–10^–6^	1.0 × 10^–6^
4	41	51 NPOE	3 MIP3	5 MWCNTs	43.0 ± 0.8	0.995	10^–4^–10^–6^	6.3 × 10^–7^
5	41	51 NPOE	3 MIP3	5 Fe_2_O_3_@MWCNTs	55.6 ± 0.3	0.986	10^–3^–10^–8^	9.8 × 10^–8^
6	41	54 NPOE	3 NIP3	5 Fe_2_O_3_@MWCNTs	49.5 ± 0.4	0.996	10^–6^–10^–4^	6.3 × 10^–7^

*An average for the values of 5 measurements, with the standard deviations of the different sensors.

**FIGURE 7:**
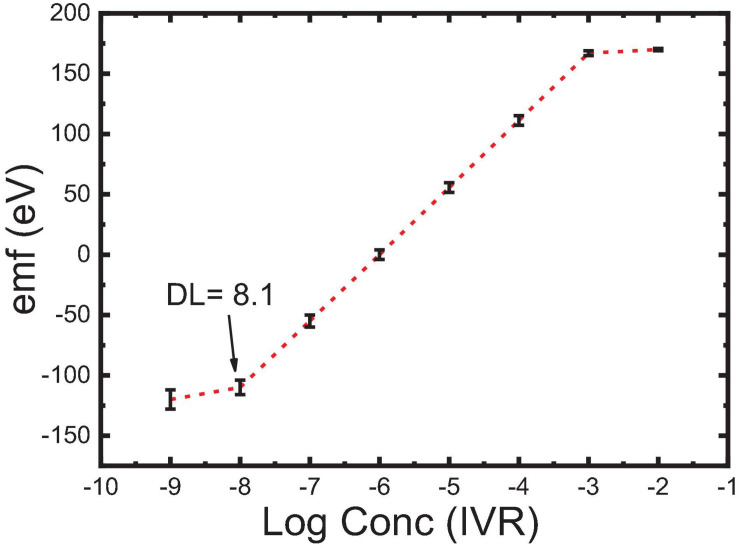
Calibration curve, concentration range, and detection limit of sensor 5, with error bars.

Type of the plasticizers (TCP and NOP) has been proved to play a key role in improving the electrochemical characteristics of the sensors. Plasticizers influence the dielectric constant of the carbon paste as well as the MIP particle’s dispensability and the transfer of the IVR drug from the solution to the electrode surface. The results showed that changing the plasticizer type from TCP (ε = 16.2) to NPOE (ε = 24.5) (Bakker et al., 1997) improved the electrode response to the sub-Nernstian value of 52.0 mV Decade^–1^, sensor 3 in Table 2. This is due to the high dielectric constant of NPOE and their relatively high molecular weight (MWt) than those of TCP. It is also possible that NPOE molecules diffuse more through (between) the MIP particles and act as shields to reduce Van der Waals forces (polymer–polymer interactive forces) and hence prevent the formation of rigid MIP aggregates (Frag et al., 2019). 1D and 2D carbon nanomaterials (Gr and MWCNTs) have been reported to be very effective when used as modifiers in sensor application (Zhang et al., 2016; Abdel-Haleem et al., 2019; Belbruno, 2019). Thus, pristine MWCNTs and Fe_2_O_3_@MWCNTs were used in electrode fabrication (5wt%) and caused a decrease in the detection limit from 1 μM for sensor 3 to 0.63 μM for sensor 4 (Table 2). This improved LOD can be related to the highly ordered structure of the tubes which can act as cylinders that increase the conductivity of the paste and improve the response at lower concentrations (Švancara et al., 2009; Zhang et al., 2016; Abdel-Haleem et al., 2018; Alam et al., 2018). It was noticed that Sensor 5 composed of graphite (41 wt%) as carbon source, MIP (3 wt%), Fe_2_O_3_@MWCNT (5 wt%), and NPOE (51 wt%) as a conductive oil exhibited a near-Nernstian value of 55.6 in a wide linear dynamic range from 1 × 10^–3^ M to 9.8 × 10^–8^ M compared to non-decorated sensors with Fe_2_O_3_@MWCNT (Figure 7).

This may be attributed to the presence of an extra amount of H-bonding interaction that exists between Fe_2_O_3_ NPs and the drug, which has been confirmed by FTIR shown in Figure 4 (Yan et al., 2013; Khorram et al., 2018). Such interaction also had an impact on the optimization of the response of sensor 5 in terms of lowering its limit of detection and increasing the linear dynamic range besides improving the slope to Near-Nernstian value (Table 2; With, 2013; Khorram et al., 2018). The comparison between sensor 5 (modified with 5wt% Fe_2_O_3_@MWCNTs) and sensor 4 (modified with 5wt% MWCNTs) indicates that this decoration increases the number of active sites available for IVR bonding. The increase in the IVR transport rate to the electrode surface leads to an improvement in the electrical conduction and a wider linear dynamic range of application (from 10^–4^–10^–6^ M to 10^–3^–10^–8^ M). This wide concentration range is suitable for the determination of IVR in overdose and lower-dose physiological samples; also, this decoration caused a decrease in the response time from 2 to 30 s (de Dios and Díaz-García, 2010; Bai and Zhou, 2014; Peterson et al., 2016).

**FIGURE 8:**
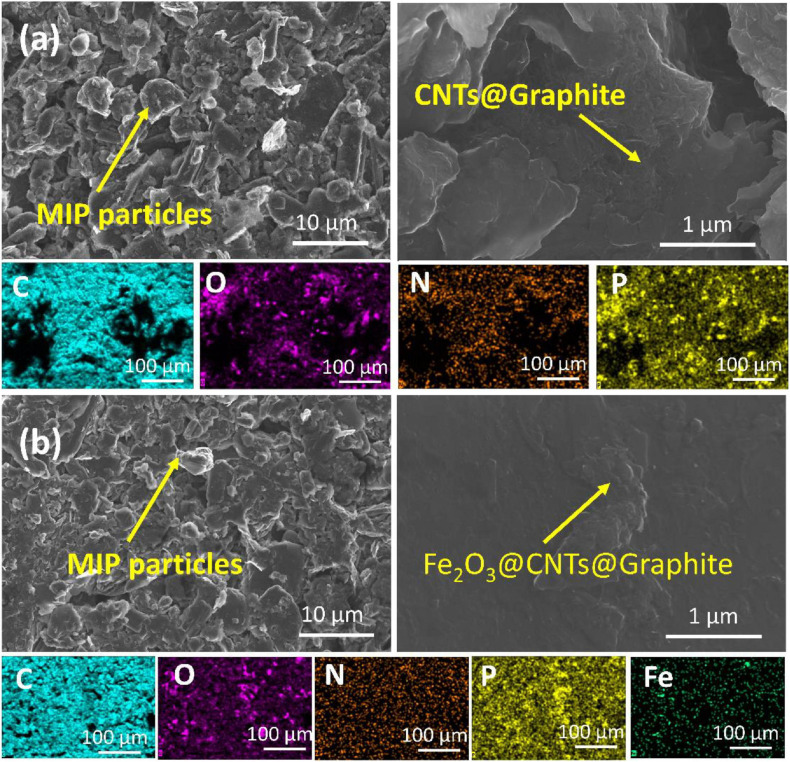
SEM images of **(a)** sensor 4 and **(b)** sensor 5 including the elemental distribution mapping in the carbon paste matrix below each.

To investigate the effect of MIP and NIP structure on sensor performance, sensor 6 was formulated in the same way and the composition of sensor 5 except NIP was used as a recognition element instead of MIP. Sensor 6 exhibited a lower response compared to sensor 5 due to the absence of the host–guest interaction as no cavities exist in NIP particles, and only H-bonding takes place on the surface of the NIP particles (Abdel-Haleem and Shehab, 2016). According to these data, sensor 5 versus sensors 4 and 6 (as a control) were further investigated in subsequent studies, i.e., SEM-EDS, selectivity against interfering species, applicability of pH range, sensor reversibility, response, and lifetime, bonding strength, and formation constant estimation. SEM-EDS elemental mapping (Figure 8) has been used to explain the improved sensing performance of sensor 5 in comparison with sensor 4 (Figure 8). The low-magnification SEM images show the higher homogeneity of the carbon paste for sensor 5 in comparison to sensor 4. Higher-magnification SEM images showed that individual as well as aggregation of MWCNTs were allocated on the surface of the graphite nanosheets. The distribution of the plasticizer, MWCNTs, and Fe_2_O_3_@CTNs within the carbon paste matrix was also studied using elemental mapping by SEM-EDS (Figure 8). The elemental mapping consistent for C, N, and O that belong mainly to graphite sheets or MWCNTs was found to be homogeneously distributed in the carbon paste of both sensors 5 and 4 (Figure 8). Fe and P homogeneity in the sensor 5 matrix indicates the uniform distribution of Fe_2_O_3_ NPs and plasticizer (TCP) in the carbon paste, respectively. This homogeneous dispersion of Fe_2_O_3_@CTN NPs leads to improvement in the limit of detection of sensor 5 compared to other sensors.

### Effect of pH

**FIGURE 9:**
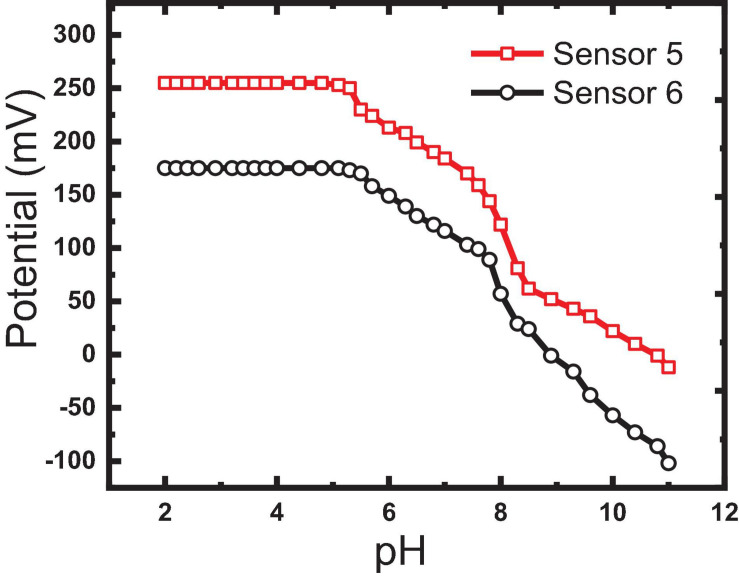
Effect of pH on the measured potential of sensors 5 and 6.

As reported before, the pH of the tested solution plays an important role in the sensitivity, detection limit, and selectivity especially in severe conditions of any electrochemical sensors (Bakker et al., 1997). The effect of pH on the investigated electrode response was tested in the pH range 2–12 by soaking the electrodes in the 10^–3^ M IVR solution and changing the pH by adding aliquots of diluted NaOH or HCl solutions, gradually. Based on the potential response obtained, sensors 5 and 6 were found to be pH-independent in the range of 2 to 5.3, as given in Figure 9. IVR exists in the mono-cationic form in this region where it contains two basic functions with pKa values of 2.4 and 8.5 (Abdel-Haleem et al., 2020). The potential decrease (from 173 to −102 mV) with the increase of the pH values (from 5.3 to 11.0) can be attributed to the ability of OH groups to form hydrogen bonding which causes interference (Bakker et al., 1997). Accordingly, no buffer adjustment was needed as the used aqueous solutions were within this stable pH range.

### Selectivity Against Interfering Ions

Selectivity is the most important feature of a MIP-based sensor to its ability to selectively bind to the target imprinted IVR drug in the presence of interfering ions that may exist in the physiological fluids and pharmaceutical preparations or may tend to form hydrogen bonding. MIP is prepared by the polymerization of functional and cross-linking monomers (MAA and EGDMA) as building monomers and AIBN as an initiator in the presence of IVR molecules (analyte drug). Removal of the IVR molecules (elution process) from the polymer matrix creates vacant recognition sites to selectively rebind to IVR molecules in a biological fluid. The resultant MIP particles possess a steric (size and shape) and chemical (spatial arrangement of complementary functional groups) memory for the IVR molecule which is able to strike binding of the closely related compounds into the available vacant recognition sites. Salt ions and large neutral molecules that exist in physiological samples or the pharmaceutical formulations were found to influence some analyst binding with MIPs (Kempe and Kempe, 2010). Thus, the selectivity of MIP for the IVR drug is depicted by the interaction of constructed sensors with different interfering species (e.g., NH_4_^+^, K^+^, Na^+^, Mg^2+^, Ca^2+^, Fe^3+^, ascorbic, lactose, glucose, maltose, dopamine, glycine) that may exist in physiological samples or the pharmaceutical formulations (Figure 9).

**FIGURE 10:**
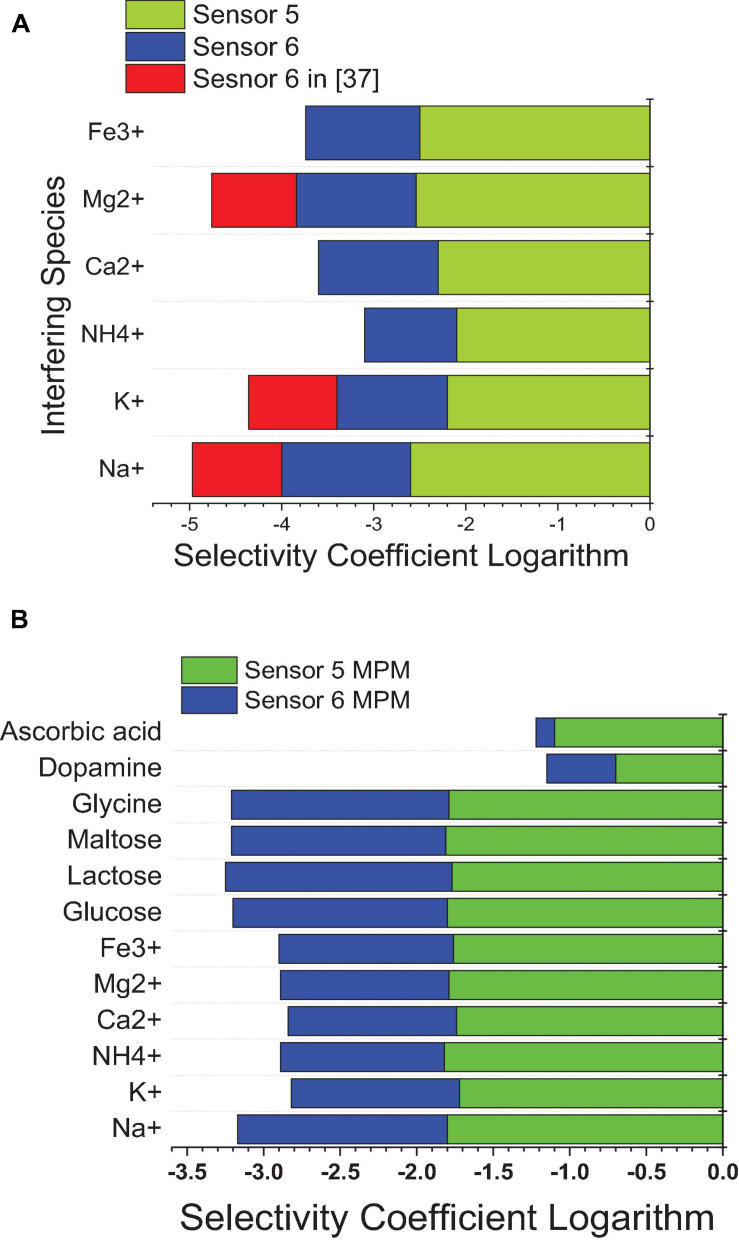
Selectivity coefficient values of sensor 5 and sensor 6 from this work against sensor 6 in reference (Abo-Talib et al., 2015) against different interfering species calculated by **(A)** the SSM method and **(B)** the MMS method.

Using SSM, better selectivity of the MIP-based electrode (sensor 5) over the NIP-based electrode (sensor 6) was noticed according to the selectivity coefficients attained. It is shown in Figure 10 that the selectivity of sensor 5 is improved over that of sensor 6 by more than an order of magnitude, and over previously reported sensor 2 (Abo-Talib et al., 2015) by about two orders of magnitude. This indicates that the interaction not only is based on hydrogen bonding as in the case of NIP3 but also depends mainly on the specific recognition binding cavities of the MIP3 selective for IVR (Hummers and Offeman, 1958; Masotti and Caporali, 2013; Bai and Zhou, 2014).

Matched potential method was performed to ensure the results of the SSM and to measure the selectivity of neutral interfering ions that cannot be measured using the SSM. The results of the MPM are lower than those of the SSM, as the instant measurement was performed for both analyte and interfering ions (Bakker et al., 1997, 2000; Abdel-Haleem et al., 2018, 2019). The improvement in selectivity was achieved by MIP-based sensors in this work over previously reported calix (Bai and Zhou, 2014) arene-based sensors (Abdel-Haleem et al., 2020) as confirmed by the lower values of the selectivity coefficient logarithm in the case of SSM and MPM; this is due to that MIP is specifically designed for the target analyte drug, and so all interfering ions are not so suited for the host–guest interaction with the MIP ionophore; thus, weaker interactions exist. In comparison to the previously reported method (Abdel Ghani et al., 2016), the proposed sensors confirmed improvement in the selectivity values (Figure 10).

### Binding Strength of IVR-MIP and Formation Constants

High-strength H-bonding between the recognition ionophore (MIP) and the analyst molecules (IVR drug) requires H-bond donors and H-bond acceptors. The functional monomers (MAA) have been used for this study because they have a low pKa = 6.5 and can be easily deprotonated (-COO^–^) within the reaction pH regime and act as H-bond acceptor. The IVR drug has amine functional groups (-NH_2_) with abundant protonated states up to a pH of 10. This also means that the IVR molecule at reaction pH can act as H-donor. IVR has two functional sites (side-chain -NH_2_ group, and -N- atom in the imidazole ring) that allow for H-bond formation. Therefore, during MIP synthesis twice the amount of MAA is added compared with the IVR molecules.

The type, numbers, and binding strength of the IVR drug to the MIP can affect the stability and shelf-life of the constructed sensor. As mentioned before, MIP3 exhibited a high affinity for strong hydrogen bonding with the IVR drug which resulted in higher stability of the sensor. The measurement of the stability constant (β_*ILn*_) is reported here for the first time in the case of MIP and NIP-based sensors using the sandwich membrane method (SMM). The results indicate that the higher value of Log β_*ILn*_ in the case of sensor 5 is based on MIP3 (11.33 ± 0.83) over sensor 6 comprising the NIP3 (2.38 ± 0.51), which can be attributed to the imprinting effect (Gurtova et al., 2013; Abdel Ghani et al., 2016; Yoshikawa et al., 2016). The size compatibility of both IVR and MIP3 ionophore aids in the formation of a larger number of H-bonds. It is noteworthy to mention that this is the first report for the quantitative determination of the extent and strength of the interaction of the MIP template.

### Sensor Reversibility

**FIGURE 11:**
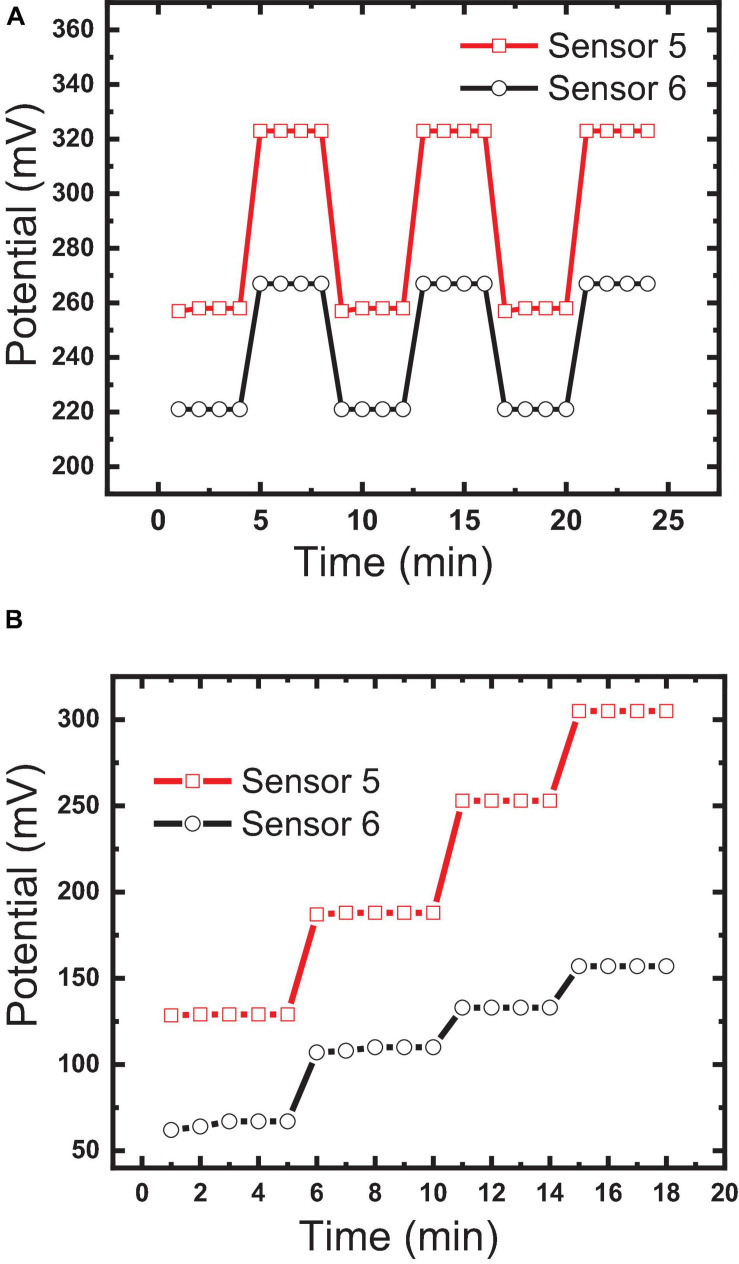
Comparison between the constructed sensor: **(A)** sensor reversibility measured by forwarding and backwarding the electrodes between two different concentrations (10^–5^, 10^–6^ M); **(B)** response time.

Reversibility measures “*the ability of the sensor to respond to the different concentrations*” (10^–3^, 10^–4^, and 10^–5^ M) in ascending and descending orders (Abdel-Haleem et al., 2018); it testifies the ability of the sensor to overcome the memory effect and to respond to different concentrations in real samples without the need for washing. Reversibility was tested for sensors 5 and 6 by measuring the potential forward and backward between two different concentrations (10^–5^, 10^–6^ M). Figure 11A shows the potential differences of 56 and 50 between the two concentrations for sensors 5 and 6, respectively, with high reversibility; this confirms that the host–guest complexation through hydrogen bond formation and the thermodynamic equilibrium phase transfer kinetics between aqueous and organic phases is fast, as previously reported by Bakker et al. (1997) and Skripnikova et al. (2017). Also, this reversibility confirms the homogeneity of the sensor surface with the absence of agglomerated sites, because agglomerated sites increase the memory effect and diminish reversibility (Bakker et al., 1997; Skripnikova et al., 2017).

### Response and Lifetimes

Response time (R_*t*_) presented as “*the time needed to reach about 90% of steady-state potential measurements*” (Bakker et al., 1997) is one of the most important characteristics that affect the applicability of the constructed sensor for the measurement of routine samples. In contrast, lifetime is the measure of the time interval in which the sensor can be used with minimum deviation in its response characteristics such as usable contraction range, Nernstian slope, and limit of detection (Bakker et al., 1997; Abdel-Haleem et al., 2018). It can be tracked by periodically performing measurements to different concentrations in the linear dynamic range.

Bakker et al. (1997) reported that potential changes at the water/electrode interface and internal diffusion are the key factors in controlling response time. The response times of sensors 5 and 6 were found to be 30 s and 1 min, respectively, as shown in Figure 10B. This fast response of sensor 5 could be explained by the strength of the host–guest interaction and size selectivity as confirmed by the high formation constant value (Bakker et al., 1997; Abdel-Haleem et al., 2019). Additionally, decoration of the MWCNTs with Fe_2_O_3_ caused a decrease in the response time, where the response time of sensor 5 was <1 min (Figure 11B). This may be attributed to the introduction of additional interactions between IVR with MWCNTs and Fe_2_O_3_ NPs (Muthukumaran et al., 2014), which is confirmed by FTIR results (Figure 4). It is also worth mentioning herein that there is a direct relationship between the IVR concentration and the response time of the sensor. The more concentration of the IVR solution leads to a longer time required for the signal to get stable. At low IVR contraction, almost all of the IVR molecules are trapped within the cavities on the MIP particle surface (faster binding). In contrast, as the concentration increases more numbers of IVR molecules require more time to permeate further into the cores of the MIP particles for binding at the available binding sites (vacant cavities) and thus increasing the response time to achieve a stable signal.

**FIGURE 12:**
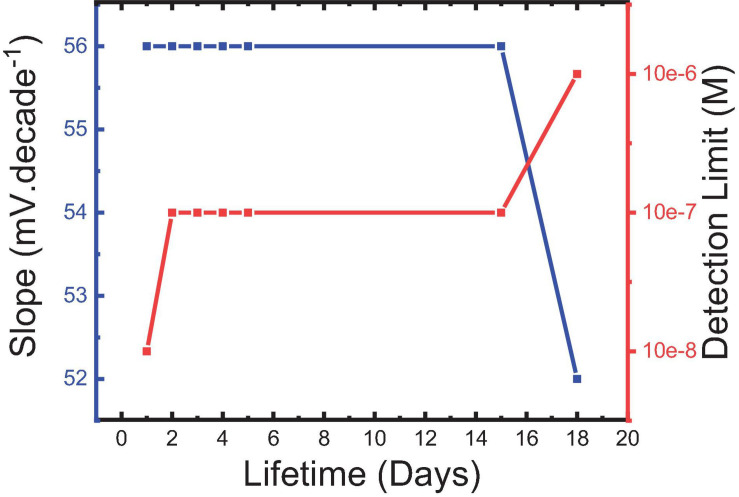
Slope of sensor 5 and its corresponding detection limit for IVR drug at the different lifetimes.

The lifetime was monitored by performing calibration curves every day and observing the different performance variables in turn. It is shown in Figure 12 that sensor 5 could be used for about 15 days with minimum deviation in its usable concentration range. Belbruno and others (Abdel-Haleem et al., 2016; Labib et al., 2016; Belbruno, 2019) confirmed the stability of the MIP particles in terms of their chemical and mechanical properties which may be a reason for the observed results. A long lifetime of MIP-based CPEs for pharmaceutical preparation with minimum deviation in the response characters for the same reason is also reported (Abdel Ghani et al., 2016).

### Applications of Sensors in Pharmaceutical and Physiological Samples

Detection and monitoring of the IVR drug in complex matrices such as spiked biological samples such as blood, serum, or plasma by sensors are very challenging where such samples contain high levels of different interfering species Na^+^, K^+^, Ca^2+^, NH_4_^+^, and Mg^2+^ and glucose, lactose, dopamine, and uric acid, which may affect the applicability of the sensor for these real samples due to the ability of these species to form hydrogen bonding with MIP ionophore.

Sensor 5 was applied for the determination of the pure IVR in spiked urine and serum samples; the recovery values were in the range of 95.0 to 106.5%, which ensures the success of this sensor for the determination of IVR in physiological samples. Also, different amounts of IVR formulation (Savapran^®^) were determined with sensor 5 and compared to the HPLC technique, the reference method reported in Nowakowska et al. (2017) and given in Table 3. The results confirmed the applicability of this sensor in pharmaceutical preparations. Measurements were performed three times, and all standard deviations were lower than one as given in Table 3, indicating the repeatability of the method. Compared with previously reported potentiometric work based on ion-pair formation (Abo-Talib et al., 2015) or ionophores (Abdel-Haleem et al., 2020) for IVR measurement, the present study showed a clear improvement in the usable concentration range when compared to the ion-pair-based sensor. In contrast, the limit of detection and selectivity coefficients were improved by one to two orders of magnitude, which highlights the role of MIP as a recognition element in the modification of CPE as given in Table 4.

**TABLE 3:** Recovery values for determination of pure IVR in spiked urine and serum samples, and results of different amounts of IVR in the commercial pharmaceutical tablets (Savapran^®^) using best electrode (sensor 5) in comparison with the reference method reported earlier (Nowakowska et al., 2017).

	Pure IVB (mg)		Savapran^®^ (mg)	
		
	Taken	Found (recovery %)	Sensor 5	Nowakowska et al., 2017
**Urine**	**1.262**	1.240 mg (99.00 ± 0.210)	1.190 ± 0.030	1.240 ± 0.060
	**0.126**	0.120 mg (97.60 ± 0.110)	–	–
**Serum**	**1.262**	1.240 mg (98.90 ± 0.190)	0.124 ± 0.019	0.118 ± 0.012
	**0.126**	0.120 mg (96.00 ± 0.140)	–	–

**TABLE 4:** Comparison between the response characteristics of the best electrode (sensor 5) of this work, with early reported sensors.

Characteristics	Abo-Talib et al., 2015, Sensor 2	Abdel-Haleem et al., 2020, sensor 5	This work, sensor 5
Nernstian slope, mV decade^–1^	58.5	58.9	56.0
Concentration range, M	10^–2^–10^–5^	10^–3^ – 10^–7^	10^–3^–10^–8^
Detection limit, M	7.8 × 10^–6^	3.6 × 10^–8^	9.8 × 10^–8^
log $K_{I\,V\,B,N\,a^{+}}^{p\,o\,t}$	−0.68	−1.79	−2.60
log $K_{I\,V\,B,K^{+}}^{p\,o\,t}$	−0.66	−1.89	−2.20
log $K_{I\,V\,B,M\,g^{2+}}^{p\,o\,t}$	−0.65	−2.17	−2.60
Response time	10 s	30 s	30 s

## Conclusion

Ivabradine is an FDA-approved drug to reduce hospitalizations for patients with symptomatic heart failure. In this study, highly sensitive and selective carbon-paste sensors have been successfully fabricated for potentiometric determination of the IVR in physiological fluids. The carbon paste electrodes are made of graphite as a carbon source, MWCNTs or Fe_2_O_3_@MWCNTs as carbon pate modifiers, tricresyl phosphate (TCP) or nitrophenyl octyl ether (NPOE) as plasticizers, and MIP as a recognition element for the IVR drug. The recognition cavities in the molecularly imprinted polymers (MIP) were suitable sites for complex IVR through H-bonding with a binding strength of (Log β_*ILn*_ = 11.33) between MIP and IVR as estimated for the first time using the sandwich membrane method. The results showed that changing the plasticizer type from TCP to NPOE and the carbon pate modifiers from MWCNTs to Fe_2_O_3_@MWCNTs significantly improved the electrode response. The best electrode (sensor 5) is formulated with 41 wt.% graphite, 5 wt.% Fe_2_O_3_@MWCNTs, 51 wt.% NPOE, and 3 wt.% MIP3. The best sensor achieved a Nernstian slope (response) of 56 mV decade^–1^ over the linear concentration range of 1.0 × 10^–3^–9.8 × 10^–8^ M and a limit of detection of 98 nM. The incorporation of Fe_2_O_3_@MWCNTs in the carbon pate increases the rate of IVR transport onto the electrode surface (carbon paste) which results in the observed decrease in the limit of detection. The designed sensor also shows high selectivity against different types of interfering species (i.e., NH_4_^+^, K^+^, Na^+^, Mg^2+^, Ca^2+^, Fe^3+^, ascorbic, maltose, glucose, lactose, dopamine, glycine), and suitability to work over a wide pH range (2–5). The constructed sensors show advantages like simplicity, high stability, automation feasibility, high accuracy, high selectivity, short response time, and applicability to blood serum, urine, and commercial formulations (Savapran^®^).

## Data Availability Statement

The raw data supporting the conclusions of this article will be made available by the authors, without undue reservation.

## Author Contributions

FA-H and AB: project administration, conceptualization, data curation, formal analysis, investigation, methodology, writing – original draft, and writing – review, and editing. EG and HE: data curation, formal analysis, investigation, and methodology. MR: formal analysis and investigation. RE: investigation, methodology, and writing – original draft. BA: data curation and formal analysis. AK: data curation, formal analysis, and methodology. All authors contributed to the article and approved the submitted version.

## Conflict of Interest

The authors declare that the research was conducted in the absence of any commercial or financial relationships that could be construed as a potential conflict of interest.
